# An Improved Apriori Association Rule for the Identification of Acupoints Combination in Treating COVID-19 Patients

**DOI:** 10.1155/2022/3900094

**Published:** 2022-09-06

**Authors:** Yanyan Zheng

**Affiliations:** Department of Computer Science and Engineering, School of Electronics and Information Engineering, Taizhou University, Linhai, Zhejiang, China

## Abstract

This work presents a data-driven method for identifying the potential core acupoint combination in COVID-19 treatment through mining the association rules from the retrieved scientific literature and guidelines for prevention and treatment of COVID-19 published all over China. It is based on the representation of the acupoint data in a binary form, the use of a novel association rule mining algorithm properly tailored for discovering the relationship of acupoint groups among combinations of different descriptions. The proposed method is applied to the real database of acupoint descriptions collected from published literature and guidelines. The obtained results show the effectiveness of the proposed method.

## 1. Introduction

In recent years, under the background of the re-recognition of the value of Chinese traditional medicine [1] and the gradual maturity of data mining technology, in order to promote the further development of traditional Chinese medicine and realize the modernization of traditional Chinese medicine, the research in the field of traditional Chinese medicine data mining is gradually active. Researchers have gradually realized the combination of data mining, machine learning, artificial intelligence, and other technologies in the research field of traditional Chinese medicine. They hope to discover the hidden principles and laws through mining, analysis, induction, and summary of a large number of clinical experience data accumulated by traditional Chinese medicine workers for thousands of years.

Since December 2019, many pneumonia cases of unknown origin have been found in many countries and regions around the world. On February 11, 2020, the disease caused by the new coronavirus was officially named coronavirus disease-19, referred to as COVID-19 [2]. The epidemic has brought serious negative effects on the global economy and society.

As a well-practiced therapeutic modality, acupuncture plays a complementary role in alleviating symptoms of certain diseases [3] and improving the health-related quality of life among COVID-19 patients [4]. It has been widely accepted that the selection and combination of acupoints are vital for successful acupuncture treatment. The principles for selecting and combining acupoints are based on the Meridian theory [5] and Biaoben theory [6] in ancient Chinese therapy.

Starting from the comprehensive consideration of the redundancy of acupuncture treatment acupoint data and the difficulty of rule mining, this paper optimizes the idea and process of the Apriori algorithm with the goal of improving the processing efficiency of the algorithm and deeply mining the association rules between acupoints, and puts forward an improved Apriori algorithm. The improved algorithm is simulated and compared with the classical Apriori algorithm to verify its effectiveness. The calculated association rule results of acupuncture point data provide an important reference basis for the analysis of acupoint combination in the treatment of COVID-19.

Section 1 presents some related work and Section 2 gives some concepts of association rules.  Section 3 describes the improved Apriori algorithm. And then, Section 4 demonstrates the case study and result of analysis. Finally, Section 5 concludes the paper.

## 2. Related Work

Apriori algorithm is a kind of association rule mining algorithm and It proceeds by identifying the frequent individual item sets in the database [7].

Apriori algorithm is widely used in many fields, for example, to explore the main influencing factors and the interaction of factors in dangerous driving conditions of urban traffic [8], the causal analysis of bridge deterioration [9], the employment trend analysis of college graduates [10], analysis of fault items in power optical transmission network [11], find frequent patterns in live transportation data [12], and the mining of association rules applied in traditional Chinese medicine are basically Apriori algorithms. For example, prescription analysis for the treatment of sleep disturbance [13], impotence [14], optic atrophy [15], and so on. Apriori algorithm is often used to analyze the combination of prescriptions and acupuncture points in the treatment of diseases by traditional Chinese medicine.  Table 1 shows the differences between relevant studies and this study.

Apriori algorithm-based association rule analysis is one aspect of association rule analysis, the other partition-based algorithm, FP-growth tree algorithm (FP means frequent pattern) were also reported. However, the two most commonly used association rule algorithms are the Apriori algorithm and the FP growth algorithm. Their respective advantages and disadvantages are rough as follows:

  Advantages of Apriori algorithm:
  ① Using a priori principle, the efficiency of generating frequent item sets layer by layer is greatly improved; [16] ② it is simple and easy to understand [17]; the data set requirements are low [18].
  Disadvantages:
  ① There are too many combinations generated in the cycle when generating the candidate item set at each step, and the elements that should not participate in the combination are not excluded [19]; ② every time the support of item set is calculated, all records in the database are scanned and compared. If it is a large database, this scanning and comparison will greatly increase the I/O cost of the computer system. And this cost shows a geometric progression increase with the increase of database records [20].

The FP growth algorithm is an association mining algorithm proposed by Han et al. for the shortcomings of the Apriori algorithm, This algorithm first compresses the database into a frequent pattern tree and then divides the compressed database into a set of conditional databases, each associated with a frequent item, and mines each conditional database separately. Finally, all mining results are aggregated to obtain a set of frequent items [21].

  Advantages:
  ① FP growth algorithm only needs to traverse the data set twice, so it is faster [22]. ② No candidate set needs to be generated [23]. ③ FP tree sorts the set in descending order of support. If different paths have the same prefix path and share storage space, the data is compressed.
  Disadvantages:
  ① The second traversal of FP tree will store many intermediate values, which will occupy a lot of memory [24]. ② Constructing an FP tree is expensive [25].

Due to the characteristics of traditional Chinese medicine data, it is suitable to use the Apriori algorithm for correlation analysis. In the field of Chinese medicine treatment, almost all association analysis algorithms used are Apriori algorithms, so this study focuses on the Apriori-based association analysis.

## 3. Problem Description and Basic Theory of Association Rules

### 3.1. Association Rules

Association rule mining is a basic data mining method used to mine interesting associations or correlations between item sets from large-scale data sets. It is very helpful for data classification, clustering, and other data mining tasks.

The formal description of association rules is as follows [26]:

Data set *D* is a collection of all things in the database. Each attribute of each record in the data set is called an item, and the collection of attributes is called an item set. Each nonempty record is called a transaction *T*.

Let *X* and *Y* be the two item sets contained in transaction *T*, that is, *X* and *Y* are both proper subsets of *T*. If *X* is a nonempty subset, *Y* is also a nonempty subset, and the intersection of *X* and *Y* is an empty set, then *X*-> *Y* constitutes an association rule in the thing set *T*.

#### 3.1.1. Support

That is to say, an association rule is an expression in the form of *X*-> *Y*, where *X* is called the preceding term and *Y* is called the following term. The probability that both *X* and *Y* are contained in the item set is called the support of *X*-> *Y*, denoted support (*X*-> *Y*) = P (*X*-> *Y*).

#### 3.1.2. Confidence

Under the condition that the prerequisite *X* of the association rule occurs, the probability that the association result *Y* occurs, that is, the probability that the item set containing *X* contains *Y* at the same time is called the confidence level of association rule *X*-> *Y,* denoted as confidence (*X*-> *Y*).

#### 3.1.3. Lift

The ratio of the possibility of including *Y* under the condition of *X* and the possibility of having *Y* in the item set without this condition is called the lift of the association rule, denoted as Lift (*X*-> *Y*) = P(*Y* |*X*)/P (*Y*) = conference (*X*-> *Y*)/P (*Y*).

#### 3.1.4. Expected Confidence

Given the association rule *X*-> *Y*, the expected confidence for the rule is the proportion of all transactions that contain item *Y*. The difference between confidence and expected confidence is a measure of the change in predictive power due to the presence of item *X* in a transaction.

Association rule mining can usually be regarded as two basic processes: ① find all frequent item sets from the transaction set, that is, find all item sets whose support is greater than the given minimum support threshold; ② use the frequent item sets found in the first step to generate all association rules, and the association rules that meet the minimum confidence are the strong association rules to be mined.

### 3.2. Apriori Algorithm

The algorithm uses a layer-by-layer search iterative method to find the largest *k*-term frequent set. First, traverse the database and search to get the candidate 1 item set and its support. If its support is lower than the minimum support, prune it to get the frequent 1 item set. Then connect the obtained frequent 1 item set to obtain the candidate 2 item sets and their support, and so on. Iterate until the frequent *K *+* *1 item set cannot be obtained, and the corresponding frequent *K* item set is the output result [27].

Apriori algorithm's a priori property is that the subset of all frequent item sets must be frequent item sets. According to the properties, a corollary is obtained that the superset of infrequent item sets must be infrequent [28, 29]. Using this property and inference, we can mine all levels of frequent item sets that meet the threshold of support and credibility.

## 4. The Improved Apriori Algorithm

### 4.1. The Idea of the Improved Apriori Algorithm

Generally, the ways to improve the process of mining frequent item sets include reducing the generation of candidate item sets and reducing the number of transaction records to be compared when obtaining item set support. Time-consuming may cause by huge item set analysis. Usually, acupoint association rule empirical analysis did not require large binary data for iteration calculation. However, as far as the algorithm itself is concerned, the algorithm is versatile. In subsequent research and expansion, higher-performance algorithms can cope with larger-scale data sets and be applied to a wider range of data.

The improved ideas are as follows:

1. Establish strong association rules, delete unrelated single transaction items, find out some association relationship between items, and mine their association. In the process of generating frequent items, Apriori algorithm needs to scan the huge transaction data set many times and delete irrelevant transaction items before and during iteration, so as to reduce the data set to a certain extent and improve the operation efficiency.
2. Row column compression through Boolean matrix to reduce the scanning times of transaction database [30–36]; In the process of scanning, the candidate item set is replaced in the form of an index table, which avoids the trouble of generating a large number of candidate item sets.
3. When searching frequent item sets and calculating confidence, trie tree is used to speed up the search. Trie tree is a data structure commonly used in data mining algorithms. This data structure occupies less memory and can quickly build and mine the effective information in the tree [37]. Many prefix tree-related technologies are applied to the algorithm of frequent item set mining to improve the execution efficiency of the algorithm. The time complexity of this algorithm is *O* (*n* log*n*).

### 4.2. Algorithm Flow

**Step 1 .**

Traverse the database once and delete irrelevant transaction item records. Set the total number of transaction items as *m* and the traversal database as. When *D*_*x*_(*x*=1,2,…, *m*)*count*=1, delete *D*_*x*_, traverse repeatedly to get a new data set *D*′.

**Step 2 .**

Get the transaction matrix and compress the rows and columns.

Convert the transaction data set *D*′ into matrix *Mat*, where transactions are sorted in column order and its *m*sets are sorted in row order. The matrix is represented as follows:

(1) $$\begin{array}{c} \begin{array}{c} \;{\;T}_{1}{\;T}_{2\;}\cdots {\;T}_{n\;}\;\\ Mat=\left[\begin{array}{c} d_{11}\;d_{11}\cdots d_{1n}\\ d_{21\;}d_{22}\cdots d_{2n}\\ \vdots \vdots \ddots \vdots \\ d_{m1\;}d_{m2}\cdots d_{mn} \end{array}\right]\begin{array}{c} I_{1}\\ I_{2}\\ \begin{array}{c} \vdots \\ I_{m} \end{array} \end{array} \end{array}\text{.} \end{array}$$

If the *i*-th item set is in the *j*-th transaction, the value *d*_*ij*_ of row *i* and column *j* of the matrix is 1, otherwise, it is 0, so the Boolean matrix is obtained.

Through the Boolean matrix obtained in the previous step, the support of the item set formed by a row in the matrix can be calculated. The support is obtained by bitwise sum operation of each row of vectors.

(2) $$\begin{array}{c} {support}_{count}=\overset{n}{\underset{j=1}{\sum }}\left(d_{i_{1}j}\cap d_{i_{2}j}\cap d_{i_{3}j}\cap \cdots \cap d_{i_{k}j}\right)\text{.} \end{array}$$

According to the Boolean matrix and the calculation method of support, the support of each set is obtained, and the item set index table is obtained. Then, the frequent item sets are obtained by comparing them with the set minimum support.

According to the nature of frequent item sets, if an item set is nonfrequent, then all supersets of the item set are also nonfrequent, which can be deleted directly, that is, row compression.

Since each transaction of Boolean matrix corresponds to a column vector, if the length of a transaction is less than *k*, it is impossible to include *k*-frequent item set *L_ k*. The transaction can be deleted directly during search, that is, column compression.

**Step 3 .**

Scan the compressed Boolean matrix again, calculate the support and create the index table. Repeat the above steps until *k*-frequent item sets can no longer be generated, and finally, get all frequent item sets in the form of an index table.

**Step 4 .**

Finally, all frequent item sets are searched in the form of trie tree to calculate the confidence, so as to generate strong association rules, that is, the association rules that users are interested in.

## 5. Case Illustration

The acupoint data of acupuncture treatment for COVID-19 are selected for experiment, and the Apriori algorithm, FP growth algorithm, and improved Apriori algorithm are compared and analyzed. The program, written in Python 3.8.3, simulates and analyzes the different values of other parameters of the algorithm, including support and confidence. According to the simulation results, the algorithm with strong applicability is selected and reasonable parameters are set for deeply mining the hidden association rules between acupoints. The simulated hardware environment is Intel (*R*) core (TM) i7-10875H CPU @2.30 GHz 16.0 GB RAM.

### 5.1. Acupoint Data

This study was conducted based on the pharmaceutical prescriptions that have achieved good preventive and therapeutic effects in practice.

We searched the treatment literature on CNKI and the official treatment plan all over China. CNKI is a key national research and information publishing institution in China. Its first database was China Academic Journals Full-text Database. In 1999, CNKI started to develop online databases. To date, CNKI has built a comprehensive China integrated knowledge resources system, including journals, doctoral dissertations, masters' theses, proceedings, newspapers, yearbooks, statistical yearbooks, eBooks, patents, and standards.

All the retrieved materials were required to include manual acupuncture, acupressure, warm acupuncture, and point application. Studies with interventions of electroacupuncture, laser acupuncture, auricular acupuncture, Acu-Transcutaneous Electrical Nerve Stimulation, sham acupuncture, single moxibustion, and Chinese herbal medicines were excluded.

The data content is the acupuncture points in COVID-19 TCM Prevention and treatment guidelines officially issued by all parts of China. A group of acupoints in the scheme are combined into a record. In these acupoint combination treatment schemes, we analyze and get the association rules between acupoints.

### 5.2. Model Building

The Apriori algorithm, FP growth algorithm, and improved Apriori algorithm model are created respectively. The different values of other parameters of the algorithm are simulated and analyzed, including support and confidence. According to the simulation results, the algorithm with strong applicability is selected and reasonable parameters are set to carry out the association rule mining of acupuncture treatment COVID-19 acupoint data.

The modeling process includes: inputting sample data and modeling parameters; compare the operation efficiency of Apriori algorithm, FP growth algorithm, and improved Apriori algorithm under different parameter settings; according to the simulation results, the algorithm with strong applicability is selected for modeling and simulation; processing the treatment acupoint database and input parameters; output the association rules between acupoints, and then analyze the results of association rules.

**Figure 1:**
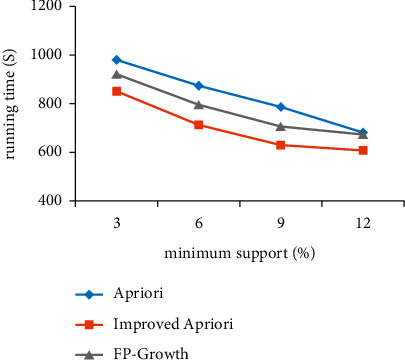
Comparison of minimum support before and after improvement.

**Figure 2:**
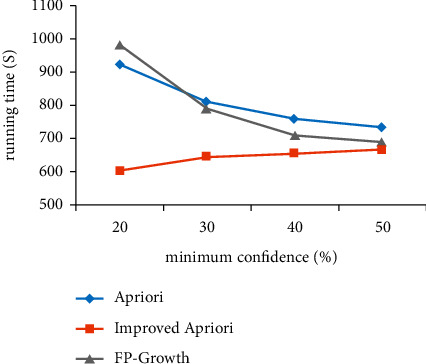
Comparison of minimum confidence before and after improvement.

Using the acupoint data set, the algorithm before and after optimization is simulated and compared with FP growth algorithm, and the variation of running time with two parameters of support and confidence is analyzed, as shown in Figures 1 and 2.

Figure 1 shows the comparison of the changes in the minimum support before and after the improvement. With the increase in support, the running time of the algorithm before and after the improvement is shortened. When the support is small, the running time of the improved algorithm is less than that of the Apriori algorithm before the optimization and FP growth algorithm. The greater the support, the more important the association rules are and the shorter the running time is.

As shown in Figure 2, the comparison between the execution time of the two algorithms before and after improvement and the change of the minimum confidence parameter is shown. With the increase in confidence, there is little difference in the running time between the two algorithms. When the confidence is small, the running time of the improved algorithm is less than that of Apriori algorithm before optimization and FP growth algorithm, and the reliability of association rules is the strongest at this time.

In conclusion, under the same database conditions and different parameter settings, it is found that the operation efficiency of the improved Apriori algorithm is significantly better than that of FP growth algorithm, and the effectiveness of the algorithm has been fully verified. Therefore, this paper applies the improved Apriori algorithm to model and simulate, and deeply mines the acupoint association rules. The minimum support of the parameter value is 13%, and the minimum confidence is 60%.

### 5.3. Algorithm Performance Verification and Result Analysis

According to the above operation results, 56 association rules are obtained (such as {cv12} = > {ST36}), which represent cv12 and ST36, that is, acupoints Zhong wan and Zusanli, the support and confidence of which simultaneous occurrence are 24% and 80%.

We extracted data on acupoints as binary data from 99 acupuncture prescriptions. As shown in Table 2, horizontal ordinate represents each traditional Chinese medicine, and the ordinate represents each prescription. It shows the distribution of traditional Chinese medicine and prescriptions.

Researchers introduced various tools from psychology and educational theories to make models explaining why games are so immersive. These approaches help us to understand the dynamics between games and players and provided a base camp for our study. Following researches suggest good points describing what is essential for good games.

**Figure 3:**
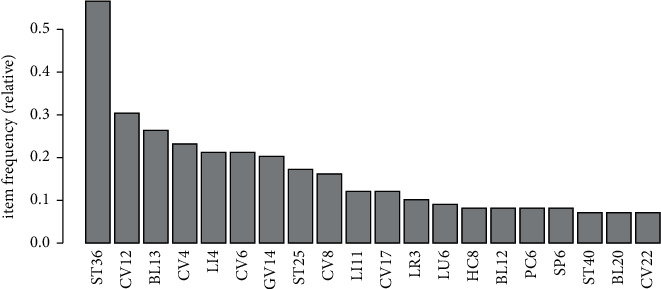
Distribution of acupoints used in the retrieved references and plans.

There were 75 acupoints extracted from the 99 retrieved acupuncture prescriptions in the literature. The acupoint frequency distribution details are presented in Figure 3. ST36, CV12, BL13, CV4, LI4, CV6, GV14, ST25, CV8, and GV14 were the top 10 frequently selected acupoints. These acupoints were frequently applied in treating respiratory diseases and relevant symptoms.

**Figure 4:**
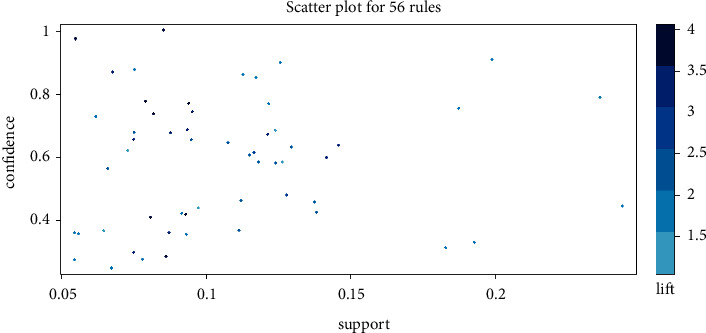
Scatter plot for 56 rules.

We investigated 56 association rules based on the integrated acupuncture data (Table 2) and the association rules were visually shown through the scatter plot. If *X* and *Y* were independent, the ratio of the observed support to that expected is called the lift. In Figure 4, the results presented that all rules had a high lift. On the support/confidence border resided the most interesting rules (sc-optimal rules). The association rules between different individual acupoints were in descending support order.  Table 3 listed the top 10 Apriori algorithm-based association rules of acupoints.

**Figure 5:**
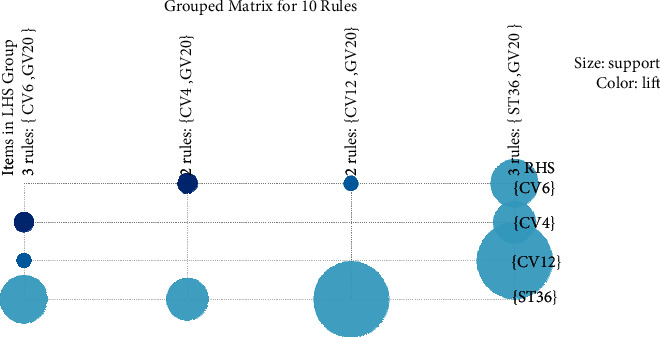
Grouped matrix for 10 association rules.

**Figure 6:**
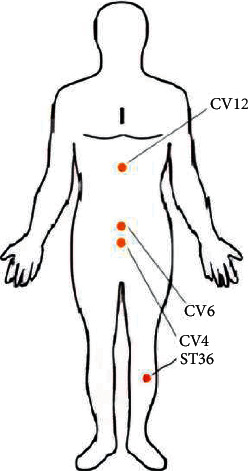
Location of the core acupoints in treating patients with COVID-19.

The grouped item sets are visualized by the graph with color and size. Based on a grouped matrix of 10 association rules, the features were visually presented (Figure 5). This plot represented clearly the association rules and was suitable for very small sets of rules to avoid cluttered presentation. Results showed that based on the evidence of the grouped matrix for 10 association rules (Figure 6), {CV12} ≥ {ST36}, {ST36} ≥ {CV12}, {ST36} ≥ {CV6}, and {CV4} ≥ {ST36} were interactively selected to reveal the rule's antecedent (LHS) and consequent (RHS) item sets. Compared with Table 3, it can be seen the interactively selected association rules were consistent for rule numbers 7 ({CV6} ≥ {CV4}), 8 ({CV4} ≥ {ECV6}), 9 ({CV17} ≥ ST36}), and 10 ({ST36} ≥ {CV17}).

## 6. Conclusion and Future Work

Aiming at the low efficiency of Apriori algorithm, an improved association rule mining model is established in this paper; mining the strong association rules between items, reducing the number of database scans, and putting forward an improved algorithm; select acupuncture data for treating COVID-19 to mine hidden association rules between acupoints and frequent acupoint combinations. The simulation results show that the improved algorithm can meet the requirements of acupoint association rule mining, improve the efficiency of data processing and the reliability of acupoint treatment COVID-19 association rule mining, and has good application value.

Besides, the algorithm used in this study may be better if the weight is considered. The next step is to consider using the weighted Apriori algorithm. In addition, the association rule analysis is one method of data mining. Later other data mining methods will be considered to combine to reflect association more comprehensively and objectively.

## Figures and Tables

**Table 1:** Comparison of publications according to their contents.

Studies	Year	Description	Field	Characteristic of algorithm
Feng et al. [8]	2022	Collect natural driving data, extract risk conditions, and analyze the direction and intensity of risk influencing factors with the confidence of association rules of apriori algorithm.	Road traffic driving	Ordinary apriori algorithm

Hu et al. [9]	2021	Apriori algorithm is used to analyze the causal association rules of bridge deterioration in Yunnan province	Bridge construction	Genetic algorithm and grey correlation analysis solve the problem of the value of support and confidence in apriori algorithm

Luo [10]	2021	Based on the scores and employment information data of higher vocational college graduates during their school years, this paper uses apriori algorithm to analyze the correlation between school performance and actual employment.	Education	Ordinary apriori algorithm

Wu and Peng [11]	2019	The power optical transmission network uses apriori algorithm to screen and retains the alarm items and fault items that occur infrequently but are actually very dangerous.	The power optical transmission network	Weighted apriori algorithm

Alan and Brant [12]	2018	Find frequent patterns in live transportation data by using association rule mining of FP growth algorithm.	Public transport ride	FP growth algorithm

Chih-Hung Lin et al. [13]	2021	Apriori algorithm is used to analyze the acupoint combination of acupuncture and moxibustion in the treatment of sleep disturbance	Traditional Chinese medicine treatment	The treatment method is acupuncture, and the disease is sleep disturbance

Tan et al. [14]	2021	Apriori algorithm is used to analyze the acupoint combination of acupuncture and moxibustion in the treatment of impotence	Traditional Chinese medicine treatment	The treatment method is acupuncture, and the disease is impotence

Zhang et al. [15]	2021	Apriori algorithm is used to analyze the acupoint combination of acupuncture and moxibustion in the treatment of optic atrophy	Traditional Chinese medicine treatment	The treatment method is acupuncture, and the disease is optic atrophy

**Table 2:** Extracted on acupoints as binary data.

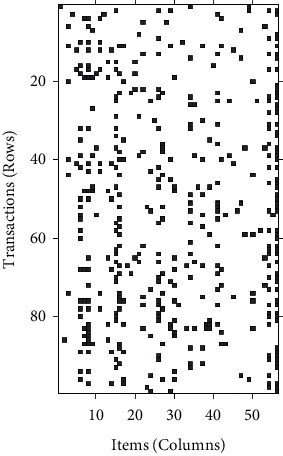

**Table 3:** Top 10 Apriori algorithm-based association rules of acupoints.

No.	LHS	RHS	Support	Confidence	Coverage	Lift	Count
[1]	{CV12}	≥{ST36}	0.2424242	0.8000000	0.3030303	1.414286	24
[2]	{ST36}	≥{CV12}	0.2424242	0.4285714	0.5656566	1.414286	24
[3]	{CV6}	≥{ST36}	0.1919192	0.9047619	0.2121212	1.599490	19
[4]	{ST36}	≥{CV6}	0.1919192	0.3392857	0.5656566	1.599490	19
[5]	{CV4}	≥{ST36}	0.1818182	0.7826087	0.2323232	1.383540	18
[6]	{ST36}	≥{CV4}	0.1818182	0.3214286	0.5656566	1.383540	18
[7]	{CV6}	≥{CV4}	0.1414141	0.6666667	0.2121212	2.869565	14
[8]	{CV4}	≥{CV6}	0.1414141	0.6086957	0.2323232	2.869565	14
[9]	{CV6}	≥{CV12}	0.1313131	0.6190476	0.2121212	2.042857	13
[10]	{CV12}	≥{CV6}	0.1313131	0.4333333	0.3030303	2.042857	13

## Data Availability

The data used to support the findings of this study are included within the article.
